# Successful treatment for a patient with antiphospholipid syndrome and decreased protein S activity exacerbated by heparin-induced thrombocytopenia: A case report

**DOI:** 10.1097/MD.0000000000040281

**Published:** 2024-11-08

**Authors:** Masahiro Nishihara, Hiroto Nagae, Shimon Otake, Shinya Asatani, Yosuke Nagasawa, Kumiko Akiya, Hirotake Inomata, Noboru Kitamura, Hideki Nakamura

**Affiliations:** a Division of Hematology and Rheumatology, Department of Medicine, Nihon University School of Medicine, Tokyo, Japan; b Department of Hematology and Rheumatology, National Hospital Organization Saitama Hospital, Saitama, Japan.

**Keywords:** anti-cardiolipin antibody, aortic thrombus, HIT, Protein S

## Abstract

**Rationale:**

Antiphospholipid antibody syndrome and protein S/C deficiency are diseases that are sometimes complicated by thrombus, and heparin-induced thrombosis (HIT) has also been reported.

**Patient Concerns:**

A male patient in his 60s with elevated D-dimer and superior mesenteric thrombus and portal vein thrombus underwent partial small intestine resection and thrombectomy. After administration of heparin, aortic thrombosis and pulmonary embolism occurred along with rapid thrombocytopenia.

**Diagnosis:**

The patient was diagnosed with HIT combined with protein S deficiency and antiphospholipid antibody syndrome.

**Interventions and outcomes:**

Heparin administration was discontinued, and plasma exchange with fresh frozen plasma replacement and argatroban administration were started. These treatments reduced D-dimer, restored platelet counts, and improved thrombosis.

**Lessons:**

Although HIT alone can cause severe arteriovenous thrombosis, our case suggests that it is important to search for the underlying procoagulant factors.

## 1. Introduction

Arterial thrombosis is generally caused by abnormalities in the arterial wall, such as arteriosclerosis obliterans or aneurysms,^[1]^ or by cardiogenic conditions, such as valvular heart disease and atrial fibrillation.^[2]^ In rare cases, may be caused by changes in the viscosity of the blood itself, such as hyperlipidemia or polycythemia vera.^[3]^ Antiphospholipid antibody syndrome and protein S/C deficiency are diseases that are sometimes complicated by thrombus,^[4]^ and heparin-induced thrombosis (HIT) has also been reported.^[5]^ Protein S acts as a cofactor for protein C, and quantitative and qualitative abnormalities in these 2 proteins are known to be a reason for the formation of blood clots.^[6]^ In either case, the anticoagulant effect of activated protein C activity is suppressed, resulting in a tendency for arteriovenous thrombosis. On the other hand, HIT is a disease that exhibits the pathology of immunogenic drug-induced thrombocytopenia, and 2 types are known.^[7]^ Type 1 HIT is not mediated by the immune system, whereas type 2 HIT is associated with arteriovenous thrombosis, and if there are multiple thrombi in the microcirculation, it progresses to disseminated intravascular coagulation. Here, we report a case of successful treatment in which the patient originally had anticardiolipin antibodies and decreased protein S activity, but subsequently showed worsening of aortic thrombosis due to the development of type 2 HIT. A review of the relevant literature is also provided.

## 2. Case report

### 2.1. Clinical course

A male in his 60s visited a local hospital on day 13 days before presenting at being referred to our institution, complaining of abdominal pain. A contrast-enhanced computed tomography (CT) scan revealed small intestinal necrosis due to superior mesenteric vein thrombosis and portal vein thrombosis, and the patient underwent partial small intestine resection and thrombectomy on the same day. Postoperatively, anticoagulation therapy with heparin was performed, but blood tests showed an increase in D-dimer levels, and contrast-enhanced CT scans showed that, in addition to the above thrombi, mural thrombi in the abdominal aorta and pulmonary artery thromboembolism had newly appeared. Thirteen days after his initial clinical visit, the patient was transported to Nihon University Itabashi Hospital for further investigation of multiple thrombosis (Day 0). The patient had hypertension, was taking amlodipine 5 mg/day, and had no family history of thrombosis. The patient had no history of drinking alcohol and had smoked 20 cigarettes per day for 25 years. Body temperature was 37.2°C, blood pressure was 132/75 mmHg, and SPO_2_ was 96% (room air). There were no abnormal heart sounds or respiratory sounds in the chest, and the abdomen was flat and soft, with no increased intestinal murmurs or tenderness. Also, no vascular stenosis sounds were heard.

A urinalysis showed no protein or occult blood and a blood test showed a normal white blood cell count at 7100 μL, hemoglobin 11.0 g/dL, and platelet count 68,000 μL. We also observed elevations in aspartate aminotransferase (36 U/L), alanine aminotransferase (67 U/L), lactate dehydrogenase (247 U/L; normal range 124 to 322 U/L), C-reactive protein (1.55 mg/dL) and serum creatinine (1.36 mg/dL) with decreased estimated glomerular filtration rate (41.8 mL/minute/1.73m^2^), although haptoglobin was within normal range. As shown in Table 1, although antithrombin-III was normal, coagulation abnormalities were observed with increases in D-dimer and fibrin degradation product. In addition, increased anti-cardiolipin IgG antibody (27.1 IU/mL), decreased total activity of protein S with a normal total amount of protein S antigen and positive anti-heparin-induced thrombocytopenia (HIT) antibody were observed (Table 1). In addition, before heparin treatment at the previous hospital, low protein S activity compared to the total amount of protein S antigen was observed. Two sets of blood culture were performed and showed negativity for HBsAg, anti-hepatitis C antibody, C7-HRP, βD-glucan and T-SPOT.

**Table 1:** Coagulation and immune system test after heparin treatment.

Test	Normal range	Results
PT (s)		17.8
APTT	27.0–45.0 s	68.7
D-dimer	<1.0 μg/mL	14.1
FDP	<5.0 μg/mL	59.7
SF	<7.0 μg/mL	140.5
PTF1 + 2	69–229 pmol/mL	468
TAT	<3.0 ng/mL	4.2
AT-III	70–130%	76
PIC	0.8 μg/mL	4.1
Total PAI-1	<50 ng/mL	23
C-reactive protein	<0.2 mg/dL	1.15
Anti-cardiolipin IgG antibody	<20 U/mL	27.1
Anti-cardiolipin IgM antibody	<20 U/mL	11.7
Anti-β2GPI IgG antibody	<20 U/mL	6.4
Anti-β2GPI IgM antibody	<20 U/mL	4.1
LA (dRVVT method)	1.2	1.2
LA (SCT method)	1.16	1.09
Protein S (total antigen)	0.8–1.31 IU/mL	1.01
Protein S (total activity)	0.86–1.18 IU/mL	0.36
Protein C (total antigen)	70–150%	67
Protein C (total activity)	64–146%	113
Anti-HIT antibody	<1.0 IU/mL	>5.0

APTT = activated partial prothrombin time, AT-III = antithrombin-III, β2GPI = β2-glycoprotein-I, dRVVT = diluted Russell‘s viper venom time, FDP = fibrin degradation product, HIT = heparin-induced thrombocytopenia, LA = lupus anticoagulant, NA = not available, PIC = plasminogen-α2plasmin inhibitor complex, PT = prothrombin time, PTF = prothrombin fragment, SCT = silica clotting time, SF = soluble fibrin monomer-fibrinogen complex, TAT = thrombin-antithrombin complex.

**Figure 1.**
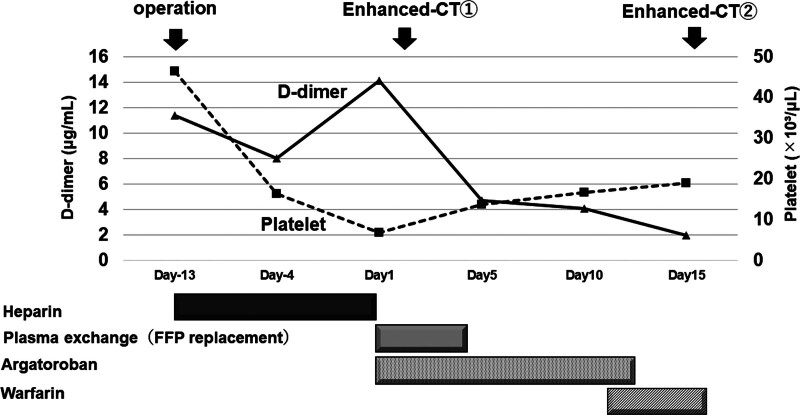
Clinical course after operation. This figure shows the changes in D-dimer and platelet numbers during treatment. FFP = fresh frozen plasma.

**Figure 2.**
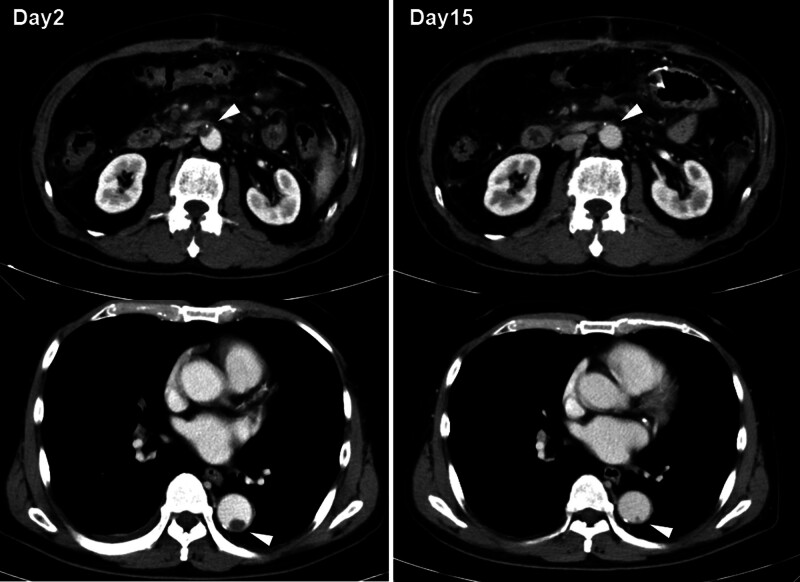
Changes in aortic thrombus before and after treatment using contrast-enhanced CT. The images show contrast-enhanced CT findings on Day 2 and Day 15. Arrowheads show the aortic mural thrombus. CT = computed tomography.

Heparin was immediately discontinued because anti-cardiolipin IgG antibody and protein S activity decreased, and because thrombocytopenia and worsening of thrombosis due to HIT were suspected (Fig. 1). When heparin was replaced with plasma exchange for 4 days and argatroban was administered for 12 days, D-dimer decreased and the platelet count recovered, and on Day 11, argatroban was replaced with warfarin. The aortic wall thrombus observed on contrast-enhanced CT on Day 2 was significantly reduced in size on Day 15 (Fig. 2). The anti-cardiolipin IgG antibody level was positive at 35.1 IU/mL even after 3 weeks, and a diagnosis of antiphospholipid syndrome (APS) was made.

## 3. Discussion

In this case, partial small intestine resection and thrombectomy were performed for a patient who presented with intestinal necrosis due to superior mesenteric thrombus, portal vein thrombosis, and portal vein blood with elevated D-dimer. Aortic thrombosis and pulmonary embolism with anti-HIT antibody-positive thrombocytopenia occurred, and anti-cardiolipin IgG antibody and decreased protein S activity were present even before heparin administration, although congenital protein S deficiency was not detected. After discontinuing heparin administration and starting plasma exchange with FFP replacement and antithrombin inhibitors, thrombosis improved.

Regarding the association between APS and protein S activity, a previous study^[8]^ found that all 94 patients in a group of patients with thrombosis had normal protein S activity, although 1 patient had protein C deficiency, suggesting that protein S deficiency is not common in APS patients with thrombosis. Todorova et al^[4]^ described that hypercoagulation in APS is closely associated with the protein C/S pathway, in which anti-phospholipid antibodies have the potential to inhibit the anti-coagulation function of protein C. In addition, they also showed that the APS patients had acquired protein C and S deficiency by showing that β2-glycoprotein-I (β2GPI) can inhibit the anticoagulation activity of protein S.

Although APS and HIT are caused by different mechanisms, they share a resemblance in that both are induced by antibodies for a protein-antigen complex and both show similar clinical features, including thrombocytopenia and arterial thrombus.^[9]^ In fact, it was reported^[10]^ that a case with both catastrophic APS and HIT was successfully treated by using thromboelastography. Coexistence of APS and HIT has also been described in a case with recurrent venous thromboembolism,^[11]^ although this case showed recurrent venous thromboembolism.

With respect to coexistence of HIT and protein S deficiency, there is a single report^[12]^ of extensive skin ulcer showing microthrombi in a patient with end stage renal disease. Compared to the frequency of APS complicated with HIT, coexistence of HIT and protein S deficiency is rare. This suggests that the microthrombi caused by decreased protein S activity are different from those caused by HIT or APS, and are also different from the pathological formations caused by antibodies against specific antigens.

A search of the literature revealed no case report in which the patient was found to be anti-HIT antibody positive and also to have decreased protein S activity and antiphospholipid antibody positivity, making this the first such report. We assume that the reason for the formation of aortic wall thrombus in this case was complex. The presence of anti-cardiolipin IgG antibodies originally poses a risk of arteriovenous thrombosis, but this combined with a decrease in protein S activity appears to have resulted in a chronic tendency to form thromboses due to a decreased ability to inhibit coagulation. Furthermore, since tests for anti-HIT antibodies became positive after heparin administration, it was assumed that excessive thrombin synthesis was enhanced.

Protein S deficiency was first reported in 1984,^[13]^ and both congenital and acquired deficiencies have since been described. It has been reported that congenital protein S deficiency occurs in 1% to 1.75% of cases of deep vein thrombosis,^[14]^ but genetic testing in this case did not identify any congenital anomalies. On the other hand, it has also been reported^[15]^ that acquired protein S deficiency is significantly more common in systemic lupus erythematosus patients than in healthy individuals. In the same report, acquired protein S deficiency was more common in the antiphospholipid antibody-positive group, and our patient was considered to have a combination of acquired protein S deficiency and anticardiolipin IgG antibody-positivity. However, it was not clear that the anti-phospholipid antibody-seropositive patients exhibited a propensity for thrombosis in the presence of acquired protein S deficiency. In this context, it is worth noting a report^[16]^ in which acquired protein S deficiency was common even in antiphospholipid antibody-positive patients without systemic lupus erythematosus. The authors of that report argued that acquired protein S deficiency itself may predispose antiphospholipid antibody-positive patients to thrombosis. In contrast, because HIT was induced by heparin administration in the present case, our patient was considered to have thrombosis exacerbated by HIT and also a pathological condition with anti-phospholipid antibody and acquired protein S deficiency. One report^[17]^ has shown that protein S deficiency in APS is an immune-mediated pathology. Therefore, the discrepancy between the antigen amount and activity is thought to be due to the inhibitory effect of anti-cardiolipin antibodies on protein S activity, rather than a mechanism that enhances antibody-dependent clearance.

This case report has some limitations. Our patient may have had a tendency to form blood clots due to infection, or a propensity for hypercoagulability due to genetic abnormalities in factor V Leiden G506A coagulation factor or prothrombin G20210A,^[18]^ which we did not search for.

In summary, we experienced a case in which APS with anti-cardiolipin IgG antibodies and decreased protein S activity caused thrombosis in multiple organs, as well as arterial wall thrombus due to HIT. Although HIT alone can cause severe arteriovenous thrombosis, our case suggests that it is important to search for the underlying procoagulant factors. In terms of treatment, it is important to search for anti-HIT antibodies, but if HIT is suspected, it is important to consider an immediate change to the treatment plan in order to save the patient’s life.

## Author contributions

**Conceptualization:** Masahiro Nishihara, Hiroto Nagae.

**Data curation:** Shimon Otake, Shinya Asatani, Kumiko Akiya, Hirotake Inomata, Noboru Kitamura.

**Formal analysis:** Hideki Nakamura, Masahiro Nishihara, Hiroto Nagae.

**Validation:** Shimon Otake, Yosuke Nagasawa, Noboru Kitamura.

**Writing – original draft:** Hideki Nakamura.
